# Genome-wide RNAi selection identifies a regulator of transmission stage-enriched gene families and cell-type differentiation in *Trypanosoma brucei*

**DOI:** 10.1371/journal.ppat.1006279

**Published:** 2017-03-23

**Authors:** Eva Rico, Alasdair Ivens, Lucy Glover, David Horn, Keith R. Matthews

**Affiliations:** 1 Centre for Immunity, Infection and Evolution, Institute for Immunology and Infection Research, School of Biological Sciences, University of Edinburgh, Edinburgh, Scotland, United Kingdom; 2 The Wellcome Trust Centre for Anti-Infectives Research, School of Life Sciences, University of Dundee, Dundee, Scotland, United Kingdom; Yale School of Public Health, UNITED STATES

## Abstract

*Trypanosoma brucei*, causing African sleeping-sickness, exploits quorum-sensing (QS) to generate the ‘stumpy forms’ necessary for the parasite’s transmission to tsetse-flies. These quiescent cells are generated by differentiation in the bloodstream from proliferative slender forms. Using genome-wide RNAi selection we screened for repressors of transmission stage-enriched mRNAs in slender forms, using the stumpy-elevated ESAG9 transcript as a model. This identified *REG9*.*1*, whose RNAi-silencing alleviated *ESAG9* repression in slender forms and tsetse-midgut procyclic forms. Interestingly, trypanosome surface protein Family 5 and Family 7 mRNAs were also elevated, which, like *ESAG9*, are *T*. *brucei* specific and stumpy-enriched. We suggest these contribute to the distinct transmission biology and vector tropism of *T*. *brucei* from other African trypanosome species. As well as surface family regulation, *REG9*.*1*-depletion generated QS-independent development to stumpy forms *in vivo*, whereas *REG9*.*1* overexpression in bloodstream forms potentiated spontaneous differentiation to procyclic forms in the absence of an external signal. Combined, this identifies *REG9*.*1* as a regulator of developmental cell fate, controlling the expression of *Trypanosoma brucei*-specific molecules elevated during transmission.

## Author summary

African trypanosomes cause important disease of humans and livestock in sub Saharan Africa and are transmitted by tsetse flies. In preparation for transmission, *Trypanosoma brucei* uses quorum sensing to generate ‘stumpy forms’ that are arrested and express a distinct subset of genes to the ‘slender forms’ that proliferate to establish the parasitaemia in the bloodstream. This necessitates that stumpy-enriched transcripts are repressed in slender forms, although the molecular control of this is unknown. Here, we have developed a genome-wide selectional strategy to isolate repressors of stumpy-enriched genes, and successfully identified a novel regulatory molecule, termed REG9.1. Silencing of REG9.1 alleviates the repression of the previously characterised stumpy-enriched ESAG9 gene family, and also two novel predicted surface protein families that are specific to *Trypansoma brucei* but absent from other African trypanosome species. REG9.1 silencing also drives density-independent differentiation to stumpy forms, whereas its ectopic expression in bloodstream forms potentiates differentiation to tsetse midgut procyclic forms in the absence of an external signal. REG9.1 is therefore identified as a novel developmental regulator whose action may contribute to the species-specific transmission biology of *Trypanosoma brucei*, which differs from that of either *Trypanosoma congolense* or *Trypanosoma vivax*.

## Introduction

African trypanosomes comprise a collection of different species *(Trypanosoma brucei spp*., *Trypanosoma congolense*, *Trypanosoma vivax)* that differ in their host range, pathology, morphology, motility and life-cycle development [1, 2]. These unicellular parasites, responsible for Human and Animal African Trypanosomiasis (HAT, AAT), are highly unusual in the eukaryota in that their genome is organised into polycistronic gene clusters in which distinct genes can show differential regulation either at the level of transcript abundance, translation or protein turnover [3].

Regulation of trypanosome gene expression is particularly important as these parasites progress through their complex life cycle [4]. For *T*. *congolense*, development is limited to the tsetse midgut and mouthparts, whereas for *T*. *vivax* the parasites are restricted to the mouthparts alone. In *T*. *brucei*, in contrast, there are several developmental forms both in the bloodstream of their mammalian host and in different compartments of the tsetse fly [5, 6]. Specifically, the parasites proliferate in the tsetse fly midgut as early and late stage procyclic forms before migration to the salivary glands where they establish as epimastigotes before developing the metacyclic forms that are mammal infective [7]. Progression through these developmental forms can be driven by the overexpression of just a single predicted RNA binding protein, RBP6, that allows the production of infective metacyclic forms from procyclic forms without an external stimulus [8]. In the bloodstream, *T*. *brucei* establish the parasitaemia as proliferative slender forms, which evade the host immune response through their expression and ability to change the expression of a dense variant-specific surface glycoprotein (VSG) coat [9–11]. As bloodstream parasite density increases, the trypanosomes exhibit a quorum-sensing like response whereupon they arrest in G1 as a quiescent and morphological ‘stumpy form’ [12]. These are competent for transmission to the tsetse fly upon their uptake in a bloodmeal where they differentiate to procyclic forms, whereas the bloodstream slender forms are killed [13]. Hence, whilst sharing the same bloodstream environment as slender forms, the stumpy form represents a distinct developmental stage, which also exhibits a distinct developmental gene expression profile [14–17]. This requires regulators that either actively maintain the slender cell form, or inhibit or promote the differentiation to stumpy forms.

Among the most predominant gene families up-regulated in stumpy forms is the *ESAG9* gene family [14, 18]. *ESAG*s (Expression Site Associated Genes) are commonly found in the polycistronic expression site locus responsible for *VSG* gene transcription. However, *ESAG9* genes are only occasional components of the *VSG* gene expression sites and several copies of the highly diverse gene family can be simultaneously expressed upon differentiation to stumpy forms [18–20]. An analysis of the predicted cell surface phylome of different African trypanosome species determined that *ESAG9* genes (included in cell surface phylome Family 2) were specific to *T*. *brucei* and missing in the related parasites *T*. *congolense* and *T*. *vivax* [21]. This was also the case for eight other cell surface phylome families, which may have functions related to the particular biology of *T*. *brucei* in their mammalian host or tsetse fly vector.

The stumpy-enriched expression of the *ESAG9* gene family is regulated through their 3’UTR [22]. Exploiting this, here we report a genome-wide RNAi selectional regimen designed to isolate repressors of stumpy-enriched gene expression operative in proliferative bloodstream slender forms. This successfully identified several negative regulators, including one (*REG9*.*1*; *REG*ulator of ESAG*9*) whose independent depletion confirmed its function as a general regulator of *ESAG9* family gene expression. Strikingly, depletion of this molecule also resulted in premature, density-independent, differentiation to stumpy forms *in vivo* and the strong upregulation of members of the *T*. *brucei* cell surface protein families 5 and 7, in addition to *ESAG9*. Each of these gene families is absent in *T*. *congolense* and *T*. *vivax*. Interestingly, ectopic expression of *REG9*.*1* at the elevated levels normally observed in procyclic forms, also caused bloodstream forms to elevate procyclic form surface protein mRNAs and potentiated differentiation to that form. This identifies *REG9*.*1* as a novel differentiation regulator and controller of the surface molecules associated with the species-specific developmental biology of *T*. *brucei*.

## Results

### A genome wide RNAi screen for repressors of molecules expressed in the *T*. *brucei* transmission stage

**Fig 1:**
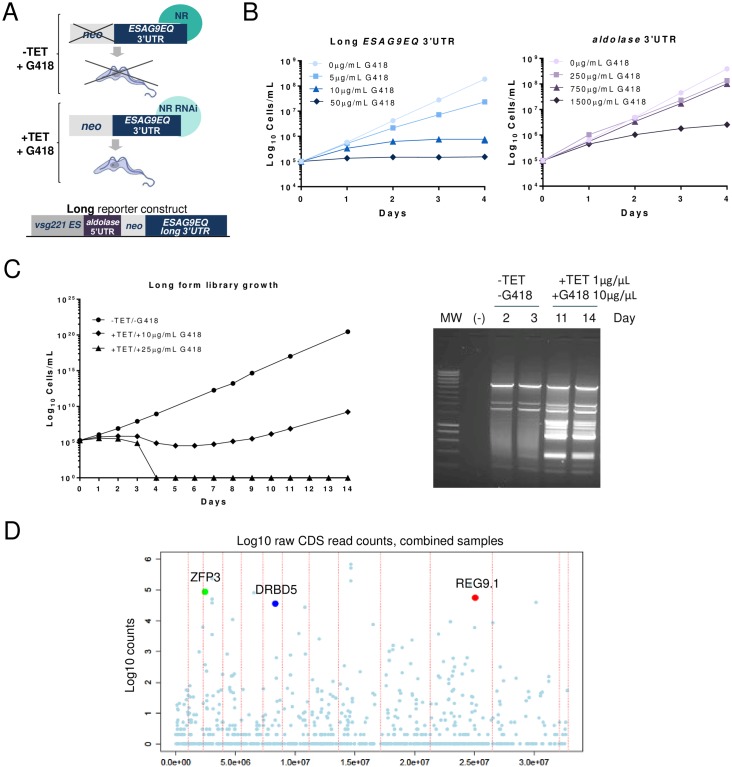
Selection of negative regulators of stumpy enriched transcript expression. A. Schematic representation of the selection schema to isolate negative regulators of *ESAG9* expression in slender forms; NR denotes a negative regulator. The selection construct used to transfect the RNAi library is shown beneath. The *NeoR* gene is flanked by the 5’UTR of the trypanosome aldolase gene, whilst the 3’UTR is derived from 1057bp downstream of the *ESAG9*-*EQ* stop codon (long form reporter, blue). B. Titration of the sensitivity of trypanosome lines with *NeoR* flanked by either the *ESAG9-EQ* 3’UTR or aldolase 3’UTR. Error bars symbolize standard deviations of biological triplicates. C. RNAi library selection of parasites containing *NeoR* flanked by the *ESAG9* 3’UTR (diamonds). The right hand panel shows the library insert amplicon profile from cells with *NeoR* flanked by the *ESAG9* 3’UTR at different stages of selection in G418 when RNAi was either induced, or not. The gel shows gDNA isolated from untreated (no tetracycline, no G418) parasites at days 2 and 3 (as a control), and treated parasites at days 11 and 14 after exposure to 1μg/ml of tetracycline and 10μg/ml G418, when parasites were outgrowing as a resistant population. D. Genome-wide distribution of selected RNAi inserts determined by ion torrent sequencing, vertical dashes representing the boundaries of each chromosome (with Chromosome 1 at the left). Targets highlighted comprise those with a high enrichment in the selected population and a predicted RNA binding domain. The full dataset is available in S1 Table.

Using genome-wide RNAi selection we sought to identify molecules that act in *T*. *brucei* slender forms to repress stumpy form enriched mRNAs. Our strategy was to generate a cell line that allowed us to select for increased resistance to G418 resulting from the loss of a repressor that acted on a NeoR gene regulated by the expression control signals of a stumpy-enriched transcript, *ESAG9* (Fig 1A). Genome-wide screens require use of the s427 monomorphic bloodstream form cell line, competent for efficient transfection with a tetracycline-inducible RNAi library [23–26]. These cells, like slender forms in natural (pleomorphic) bloodstream infections, express little *ESAG9* mRNA due to posttranscriptional repression [18, 22]. To carry out the screen, we initially transfected the s427 monomorphic parental cell line with a construct comprising the neomycin resistance (*NeoR*) gene flanked downstream by a 1057nt fragment containing the previously characterised 3’UTR control signals and intergenic sequence of the *ESAG9* gene Tb927.5.4620 [22] (‘*ESAG9-EQ long 3’UTR’* in Fig 1A). When the resulting cell line was assayed with a titration of G418, cells transfected with the *NeoR* gene coupled to the *ESAG9* flanking sequence were killed at ≥10μg/ml G418, whereas cells transfected with the same construct but where the *NeoR* gene was flanked by the constitutively-expressed aldolase gene 3’UTR were viable at >750μg/ml G418 (Fig 1B). This confirmed our previous analyses demonstrating that the *ESAG9* downstream sequences repress gene expression in proliferative slender forms [22].

The cell line with the construct comprising the *NeoR* gene flanked by the *ESAG9* intergenic sequence was then stably transfected with the tetracycline inducible genome-wide RNAi library [25]. Thereafter, uninduced and induced populations were selected with 10μg/ml G418 (Fig 1C); eventual outgrowth of surviving cells occurred at 10μg/ml G418 with RNAi induction but not at 25μg/ml G418. In contrast, in the absence of G418 or tetracycline, growth was maintained unimpeded through the duration of the experiment (Fig 1C). After genomic DNA isolation and analysis of the RNAi insert amplicons from parasites throughout the selection period, a significant change in the amplicon profile was observed with strong enrichment of discrete bands after selection for 11 and 14 days in G418 (Fig 1C, right hand panel).

**Fig 2:**
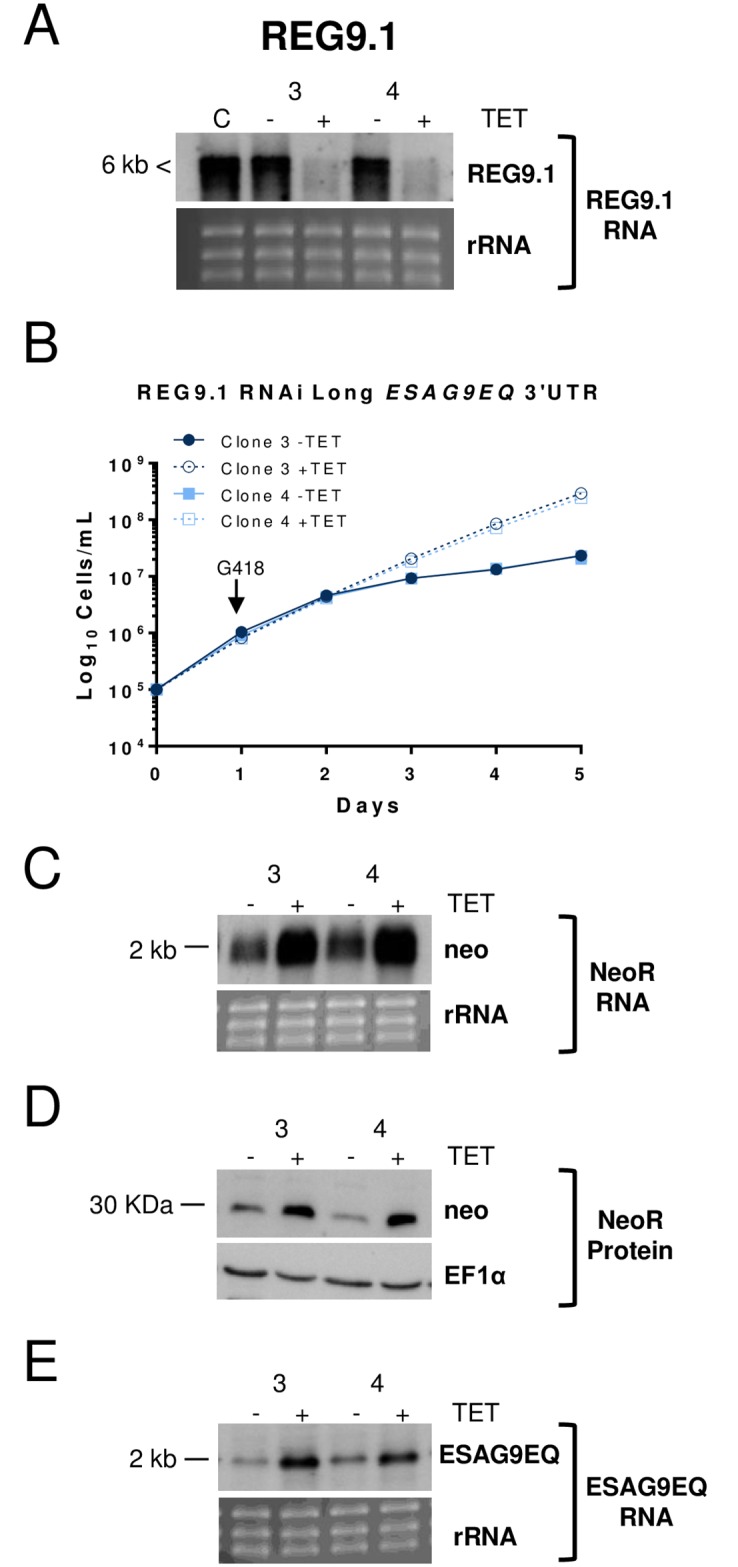
Validation of RNAi lines silencing REG9.1 selected from the RNAi library. A. Northern blot demonstrating the inducible gene silencing of *REG9*.*1*. Two independent RNAi clones were analysed (*REG9*.*1*, clone 3 and 4).); rRNA was used as a loading control. Smearing is caused by the transcript being larger than 6 kb. B. G418 resistance (at 10μg/ml) of the respective RNAi clones when RNAi was induced or not. RNAi resulted in decreased sensitivity to G418. Error bars (obscured by the symbols) symbolize standard deviations of the triplicate assays of each clone ±tetracycline. C. Northern blot of *NeoR* transcript levels when each *REG9*.*1* RNAi line was induced or not; rRNA was used as a loading control. D. Western blot of *NeoR* protein when each REG9.1 RNAi line was induced or not; EF1α was used as a loading control. E. Northern blot of *ESAG9*-*EQ* transcript levels when each REG9.1 RNAi line was induced or not; rRNA was used as a loading control.

To analyse the selected material in detail, ion torrent deep sequencing [12] was carried out on replicate samples of the day 14 selected populations. The resulting reads were mapped against the *T*. *brucei* v9 genome assembly (Fig 1D; S1 Table) and the top 30 enriched loci analysed for alignment to genes with a predicted role in mRNA regulation. This identified three candidate genes, of which two were already characterised to some extent (Tb927.6.3480, *DRBD5*,[27]; Tb927.3.720, *TbZFP3*; [28, 29]) and a novel gene encoding a hypothetical protein (*REG*ulator of *ESAG9-1*, *REG9*.*1*; Tb927.11.14220) (Fig 1D). To verify the screen outputs, independent RNAi constructs derived from each candidate gene were then transfected into the monomorphic reporter cell line containing the *NeoR* gene flanked by the *ESAG9* intergenic region. *NeoR* protein expression was increased upon silencing of each potential regulator, validating the selection of each candidate, but RNAi targeting *DRBD5* and *ZFP3* did not elevate endogenous *ESAG9* transcript (S1 Fig). In the case of DRDB5, this was because the 5’UTR of *ESAG9* contributes to ESAG9 repression in addition to the 3’UTR (Rico and Matthews, personal observations). In contrast, *REG9*.*1* knockdown (Fig 2A) resulted in enhanced G418 resistance (Fig 2B), elevation of *NeoR* mRNA (Fig 2C) and protein (Fig 2D), and elevation of endogenous *ESAG9-EQ* transcripts (Fig 2E) in monomorphic cells.

To explore the regulation of the *ESAG9-EQ* mRNA further, *REG9*.*1* RNAi was carried out in reporter lines harbouring *ESAG9-EQ* downstream sequences extending only 400 nt from the stop codon (*ESAG9*-‘short form’), or lacking a previously mapped regulatory element contributing to stumpy-enriched expression (*ESAG9* ΔE)(S2A Fig). The *ESAG9*-short form 3’UTR was less sensitive to G418 than the control reporter with 1057 nt of downstream sequence, consistent with the deletion of repressive elements from the 3’UTR (S2B Fig). Nonetheless upon *REG9*.*1* depletion, both the short form 3’UTR and the ΔE mutant exhibited increased NeoR mRNA, demonstrating the retention of *REG9*.*1*-dependent regulatory motifs within each 3’UTR (S2C and S2D Fig). Thus, REG9.1 negatively regulates *ESAG9* through downstream sequences, but this was not simply restricted to discrete proximal or distal sequences in the *ESAG9* intergenic region, nor a small region of the 3’UTR.

Independent RNAi lines for 10 potential further targets identified in the library screen were also generated but these either exhibited no effective RNA knockdown, did not enhance G418 resistance upon RNAi or generated a strong growth phenotype precluding informative follow-up studies (S1 Table).

### *REG9*.*1* is differentially expressed

**Fig 3:**
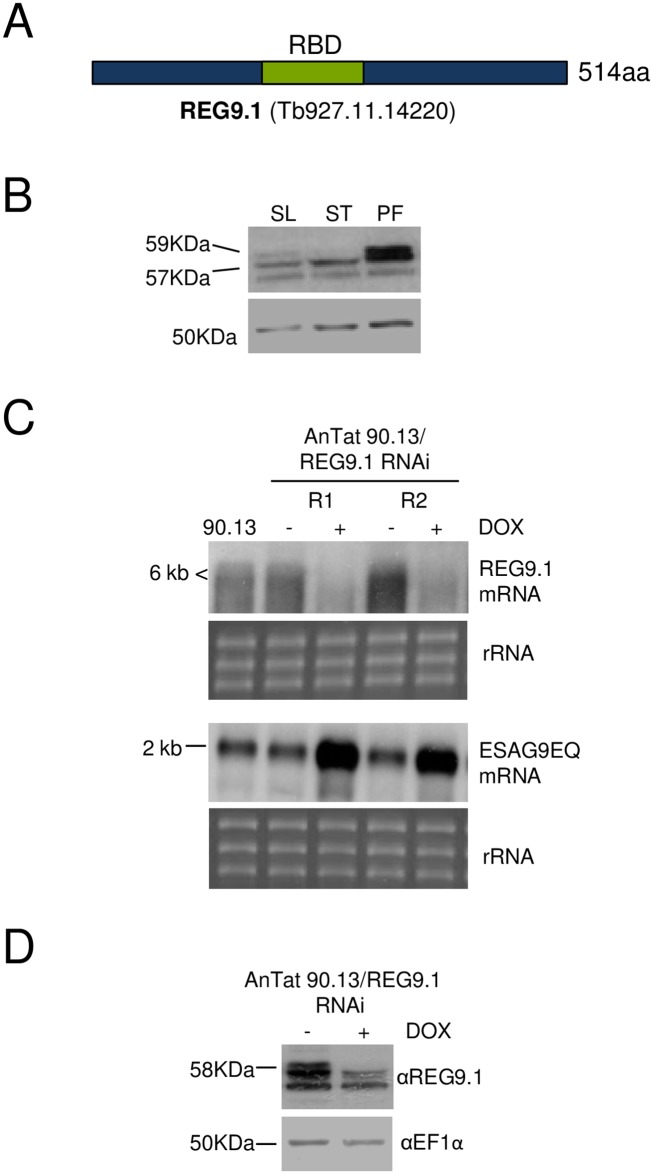
REG9.1 developmental expression profile and REG9.1 RNAi in pleomorphic parasites. A. Schematic representation of REG9.1 showing the position of its single predicted RNA binding domain (RBD). B. Protein expression of REG9.1. The antibody detects three bands, the lower band being detected inconsistently between blots. The abundance of the two upper bands differ in different life cycle stages, with the uppermost band being less abundant in stumpy forms. Procyclic forms express more REG9.1 than bloodstream forms. C. Northern blot for *REG9*.*1* and *ESAG9*-*EQ* in the parental line (*T*. *brucei* AnTat90:13) or two replicates of the pleomorphic RNAi lines induced or not to silence *REG9*.*1*. Smearing is caused by the REG9.1 transcript being larger than 6 kb. rRNA provides the loading control. *ESAG9-EQ* mRNA is elevated upon *REG9*.*1* depletion in each replicate. D. Western blot of REG9.1 expression in pleomorphic cells induced to deplete expression via RNAi. The loading control was an antibody detecting EF1α.

*REG9*.*1* is predicted to encode a 54.4kDa protein with an acidic PI (pH 5.5) and a predicted RNA-binding motif (Superfamily SSF54928 domain)(Fig 3A). In a previous mRNA tethering screen, direct binding in parasites of a *REG9*.*1*-lambda N fusion protein to a reporter construct mRNA with BoxB binding sites repressed expression, supporting a potential regulatory function for *REG9*.*1* [27].

An antibody was generated against the REG9.1 protein and the developmental expression profile of the protein assessed in pleomorphic slender and stumpy forms, and cultured insect procyclic forms (Fig 3B). In addition to a band at 54 kDa, which was inconsistently detected, two bands were detected at 57kDa and 59kDa potentially representing different post-translationally modified forms of the protein (Fig 3B). The upper band was somewhat less abundant in stumpy forms compared to slender and procyclic forms, and in procyclic forms the overall abundance of both 57 and 59 kDa bands was greater than in either bloodstream form. Consistent with this, quantitative proteomic analysis has previously indicated that *REG9*.*1* is 2- [17] to 3-fold [30] more abundant in procyclic forms vs. stumpy forms.

To further explore the function of *REG9*.*1*, pleomorphic cell lines were generated that either silenced this gene by RNAi or inducibly overexpressed the protein using the pLEW ectopic expression vector [31]. Pleomorphic cells are considerably more difficult to analyse and genetically manipulate than laboratory adapted monomorphic lines but are necessary for developmental studies since they undergo natural progression to stumpy forms, which is associated with physiological *ESAG9* regulation. Fig 3C shows northern analysis of two independent pleomorphic RNAi replicates demonstrating effective depletion of *REG9*.*1*. Matching the original selection in monomorphic cells, in both cases, *REG9*.*1* depletion caused upregulation of *ESAG9*-*EQ* mRNA *in vitro*. In separate samples, the protein levels of *REG9*.*1* were also correspondingly reduced upon RNAi (Fig 3D).

**Fig 4:**
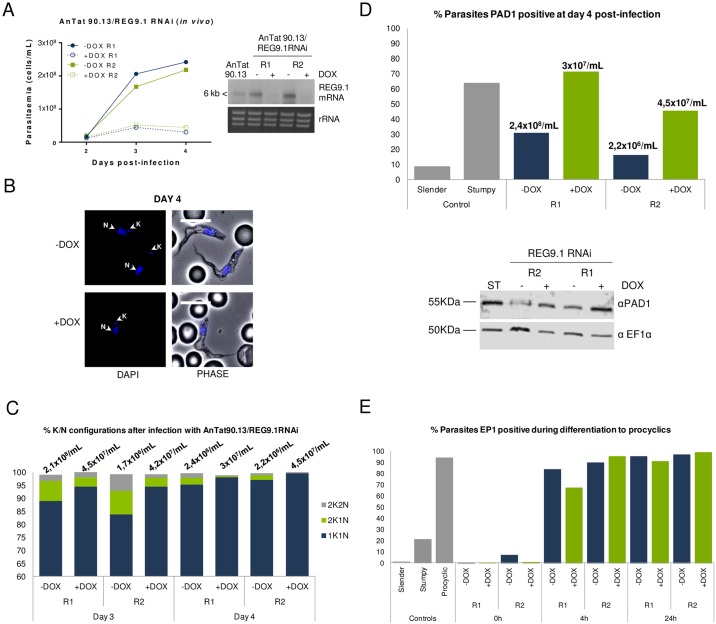
Phenotype of *REG9*.*1* gene silencing *in vivo*. A. Growth of *REG9*.*1* pleomorphic RNAi lines *in vivo*, with RNAi induced or not by doxycycline. The inset shows the respective abundance of *REG9*.*1* transcript in each case after cells were harvested on day 4 post infection and RNA prepared. Two mice replicates (R1 and R2) were used for each condition (–DOX and +DOX). B. Morphology of cells on day 4 of infection. Two cells are shown for the uninduced population, both being ‘intermediate’ in morphology, although the upper cell is somewhat more stumpy with the nucleus more proximal to the kinetoplast; in the induced population a stumpy cell is shown, with the nucleus moved toward the cell posterior. Hence although at a much lower parasitaemia (see panel A), the stumpy morphology of induced cells was equivalent to, or greater than, uninduced cells. The DAPI panel at the left hand side shows the positions of the nucleus (N) and kinetoplast (K) in each case Bar = 10μm. C. Cell cycle profile of pleomorphic RNAi lines (R1, R2) induced or not to silence *REG9*.*1* expression. The kinetoplast/nuclear configuration was assessed on day 3 and day 4 of infection, with the associated parasitaemia shown above each bar. At least 250 cells were counted for each condition/replicate/day. D. PAD1 expression profile determined by flow cytometry in the *REG9*.*1* RNAi lines (R1, R2) induced or not, with slender or stumpy *T*. *brucei* AnTat1.1 cells as controls. The parasitaemia of each population at harvest is shown. PAD positivity was determined by gating with respect to the Slender (negative) and Stumpy (positive) control samples. Below the chart is the PAD1 expression profile determined by western blotting in the *REG9*.*1* RNAi lines (R1, R2) induced or not, when harvested, with stumpy AnTat1.1 cells as control (‘ST’). EF1α abundance was used as a loading control. E. Flow cytometry of the expression of EP procyclin during differentiation to procyclic forms of each *REG9*.*1* RNAi line (R1, R2) 0h, 4h and 24h after exposure to 6mM cis aconitate. EP procyclin expression on slender, stumpy and procyclic forms provide controls. A low level of EP procyclin expression is detected on stumpy cells, this being induced during isolation and cell preparation procedures. Although at lower parasitaemia (panel A) the induced cells differentiate as effectively as uninduced cells demonstrating the physiological relevance of the stumpy forms generated.

To assess the phenotype resulting from *REG9*.*1* depletion *in vivo*, the pleomorphic RNAi line was inoculated into mice, with gene silencing being induced by provision of doxycycline to the drinking water of replicate infected animals 1-day post infection. Strikingly, this resulted in a rapid cessation of the increasing parasitaemia such that induced parasites remained below 5x10^7^ parasites/ml compared to uninduced parasites that reached 2.5x10^8^ parasites/ml (Fig 4A). Despite their low parasitaemia, the morphology of the induced parasites resembled intermediate/stumpy forms generated by uninduced parasites at high parasitaemia after 4 days of infection (Fig 4B; S3A Fig). To determine whether these had the expected physiological characteristics of stumpy forms, we tested their cell cycle arrest in G0/G1, their expression of the stumpy specific marker protein PAD1 [32] and their capacity for differentiation to procyclic forms upon harvest and exposure in culture to 6mM cis aconitate. Fig 4C shows that the induced parasites accumulated with a 1 kinetoplast 1 nucleus configuration (consistent with G1/G0 arrest) and expressed PAD1 as assessed by both flow cytometry and western blotting (Fig 4D), despite being at 5-10-fold lower cell density than uninduced parasites. Further, the isolated parasites were able to differentiate synchronously to procyclic forms, expressing the procyclic form surface protein EP procyclin on >60% of cells 4h after exposure to 6mM cis aconitate (Fig 4E). The cells also re-entered a proliferative cell cycle after 24 h in these differentiation conditions (S3B Fig). All of these assays demonstrated that *REG9*.*1* depletion drives cells to generate physiologically valid stumpy forms at much lower parasitaemia than when uninduced, overriding normal quorum sensing mechanisms.

### *REG9*.*1* regulates mRNAs for developmentally regulated cell surface proteins

**Fig 5:**
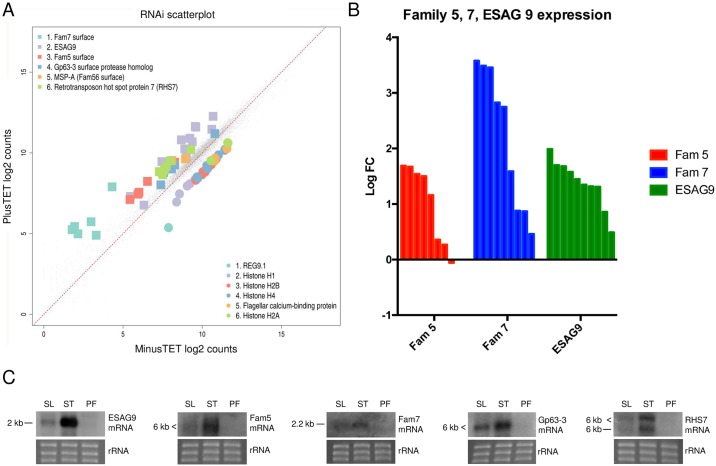
Global transcriptome changes associated with *REG9*.*1* RNAi. A. Transcripts up and down regulated upon *REG9*.*1* RNAi in pleomorphic trypanosomes. The data represents an analysis of biological replicates whether cells were induced or not to deplete *REG9*.*1* (two replicates each). The down regulation of histones and the upregulation of key regulated molecules, including *ESAG9* (lilac), Family 5 (red) and Family 7 members (blue) is highlighted. B. Relative LogFC (Log2 fold change) expression of different members of *T*. *brucei* surface protein Family 5 (red) Family 7 (blue) and ESAG9 (green) after *REG9*.*1* depletion by RNAi. C. Northern blots of *ESAG9*, Family 5 and family 7 transcripts in slender (SL), stumpy (ST) and procyclic forms (PF). The similarity within family 5 and 7 results in the detection of multiple family members by the riboprobes, generating a smear of bands. Northerns for GP63 and RHS7 are also shown, both being upregulated in the RNA-seq dataset. rRNA provides the loading control.

The wider consequences for the parasites’ transcriptome of silencing *REG9*.*1* were then examined. Thus, *in vitro* grown biological replicates of the pleomorphic RNAi line, induced or not for 48h, were analysed by RNA-seq (Fig 5; S2 Table). Fig 5A and 5B demonstrate that not only *ESAG9-EQ* showed elevated expression but also many other members of the *ESAG9* family (lilac squares in Fig 5A; Green bars in Fig 5B), including those with only distant relationship to *ESAG9-EQ*, demonstrating co-regulation of the family. Interestingly, members of other gene families were also elevated and coregulated with *ESAG9* upon *REG9*.*1* depletion. This included members of the cell surface phylome Family 5 (red squares, Fig 5A) and Family 7 (blue squares, Fig 5A) both of which, like *ESAG9*, are restricted to *T*. *brucei*, being absent from the genomes of *T*. *congolense* or *T*. *vivax* [21]. Family 5 has 8 members, of which 5 were elevated upon *REG9*.*1* depletion (Fig 5B, red bars); Family 7 comprises 9 members of which 6 were > log2FC elevated in the absence of *REG9*.*1* (Fig 5B, blue bars). Northern analysis using riboprobes simultaneously detecting several members of these surface protein family mRNAs demonstrated their elevated expression in stumpy forms, matching the expression profile of *ESAG9* (Fig 5C). Hence *REG9*.*1* acts as a negative regulator of at least three *T*. *brucei* specific surface protein families each of which is expressed in the parasite’s transmission stage. Apart from these families, other upregulated transcripts also encoded predicted surface proteins (e.g. MSP-A, Gp63; Fig 5A and Fig 5C), whereas down-regulated transcripts were related to cell proliferation and structure (e.g. histones, flagellar and paraflagellar rod proteins) (Fig 5A). Transcripts for the PAD proteins, diagnostic for stumpy forms, were not significantly regulated (S2 Table).

**Fig 6:**
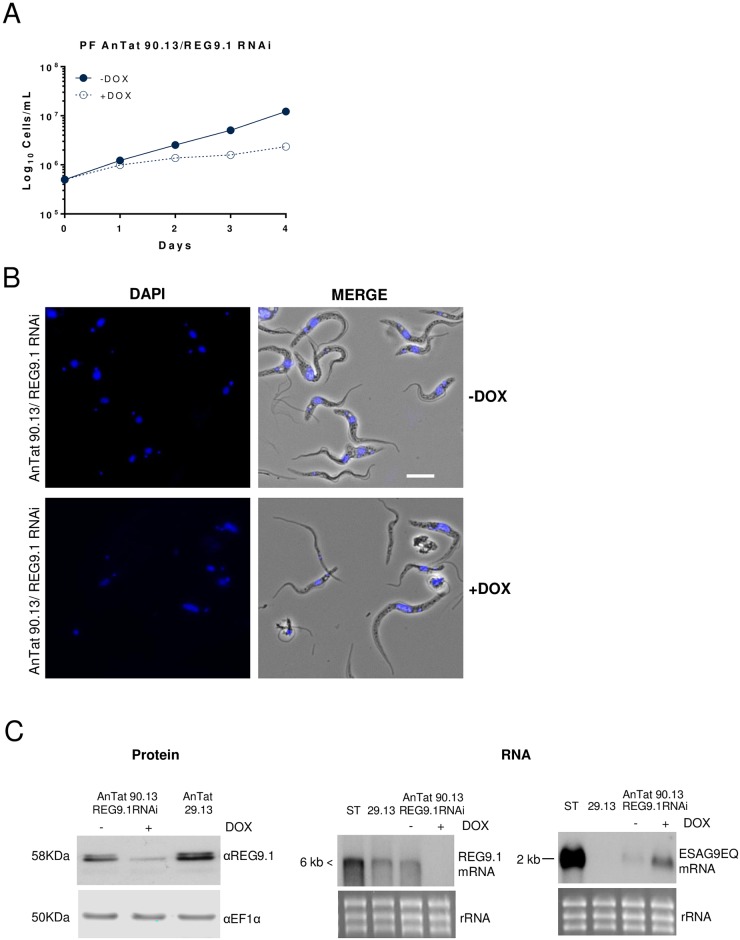
*REG9*.*1* RNAi in procyclic forms. A. Growth of procyclic form AnTat1.1 90:13 RNAi lines induced and uninduced to deplete *REG9*.*1*. B. Morphology of *T*. *brucei* AnTat 90:13 procyclic form *REG9*.*1* RNAi lines, induced and uninduced. DAPI stained and phase-contrast/DAPI stained cells (‘Merge’) are shown. Bar = 10μm. C. On the left panel is shown a Western blot of REG9.1 in AnTat1.1 90:13 procyclic forms whether induced or not to deplete *REG9*.*1* transcript by RNAi for 96 h. Established *T*. *brucei* AnTat 29:13 procyclic forms were used as a control; the loading control was an antibody detecting EF1α. On the right are northern blots of *REG9*.*1* and *ESAG9-EQ* mRNAs in stumpy forms, procyclic AnTat1.1 29:13 control cells and the AnTat1.1 90:13 procyclic form *REG9*.*1* RNAi line. Upon *REG9*.*1* depletion (for 48h), *ESAG9-EQ* levels are elevated. Smearing for *REG9*.*1* is caused by the transcript being larger than 6 kb.

To investigate whether the transcript regulation observed simply represented a consequence of the *REG9*.*1* RNAi cells developing toward a stumpy phenotype and thereby upregulating stumpy-enriched transcripts, we analysed REG9.1-depletion in procyclic forms, which do not normally express *ESAG9* transcripts. Thus, the uninduced pleomorphic bloodstream form REG9.1 RNAi line was differentiated with 6mM cis aconitate and, once stably established as procyclic forms, *REG9*.*1* depletion was induced with tetracycline. The induced cells maintained growth, albeit at a reduced level compared to uninduced cells (Fig 6A), and the cells appeared elongated and the population contained many anucleate cytoplasts (zoids; [33]) (Fig 6B). Western blotting confirmed that the REG9.1 protein was effectively depleted in the induced population (Fig 6C), where many of the cells also showed severely reduced motility, accumulating at the bottom of the culture flask, contrasting with the uninduced population that remained distributed throughout the medium (S1 and S2 Supplementary data). Nonetheless, when RNA was analysed in the induced populations, *ESAG9-EQ* transcripts were found to be upregulated (and to a limited extent in uninduced cells where *REG9*.*1* RNAi was somewhat leaky), consistent with the observations in bloodstream forms. This eliminated the regulation of these mRNAs in bloodstream forms being a simple indirect consequence of induced stumpy formation and further supported a function for *REG9*.*1* in the repression of stumpy-enriched mRNAs regardless of life cycle stage.

### Ectopic overexpression of *REG9*.*1* potentiates differentiation to procyclic forms

**Fig 7:**
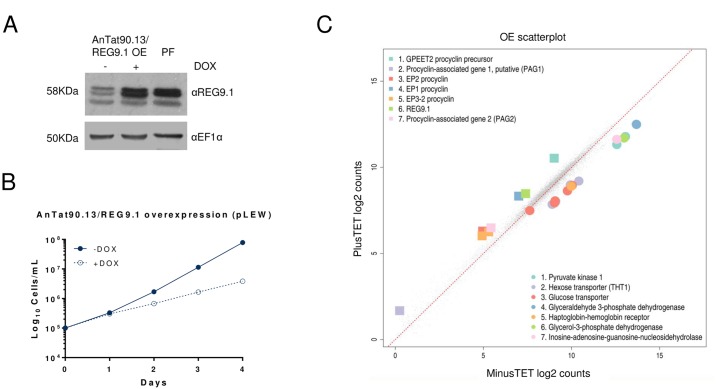
Pleomorphic trypanosomes expressing *REG9*.*1* ectopically using pLEW express procyclic form mRNAs. A. Ectopic overexpression of *REG9*.*1* in pleomorphic bloodstream forms. The panel shows the expressed REG9.1 detected with an antibody to the protein in bloodstream forms (induced or uninduced for overexpression using pLEW), with procyclic form (PF) samples included to demonstrate that similar levels of expression to procyclic forms were generated upon induction. *B*. *In vitro* growth of parasites induced to express *REG9*.*1* from the pLEW expression vector. C. Transcripts up and down regulated upon *REG9*.*1* ectopic expression using pLEW in pleomorphic trypanosomes. The data represents an analysis of two biological replicates whether cells were induced or not to ectopically express *REG9*.*1*.

**Fig 8:**
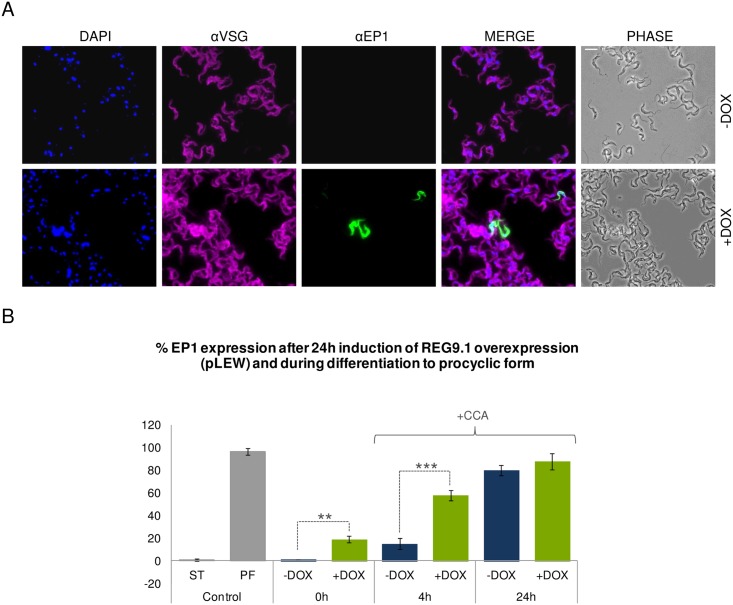
Ectopic expression of REG9.1 in pleomorphic trypanosomes potentiates differentiation. A. Immunofluorescence showing EP procyclin expression on pleomorphic parasites induced to express *REG9*.*1* ectopically using pLEW. αVSG AnTat1.1 is used as a marker of the bloodstream forms surface. B. Flow cytometry showing EP procyclin expression on pleomorphic slender parasites induced to express *REG9*.*1* ectopically using pLEW. 24h after induction parasites were resuspended in procyclic form culture medium (SDM79) and induced to form procyclic forms in the presence of cis aconitate (+CCA). Induced cells differentiated faster than the uninduced (**, P<0.01; ***, P<0.001).

To assess whether an elevation of *REG9*.*1* levels generated converse regulatory effects to that of RNAi, and so delayed stumpy formation, pleomorphic bloodstream cells were generated that were able to overexpress *REG9*.*1*. Supporting the physiological relevance of the *REG9*.*1* ectopic expression generated, the level of *REG9*.*1* protein in bloodstream forms generated using the pLEW expression vector was equivalent to the level normally seen in procyclic forms (Fig 7A). The induced bloodstream form parasites remained viable and continued to proliferate, albeit at a slightly reduced level (Fig 7B). However, analysis of the global transcriptome dataset for REG9.1 overexpression did not generate the opposite regulatory profile to RNAi. Rather, in this case bloodstream-enriched mRNAs were downregulated and procyclic-form enriched transcripts were elevated, particularly those encoding the procyclic form surface proteins EP and GPEET procyclin (S3 Table; Fig 7C). This suggested a shift to a procyclic form-like transcriptome profile upon REG9.1 overexpression. To determine if this was also reflected at the protein level, bloodstream forms overexpressing REG9.1 were analysed for EP procyclin expression by immunofluorescence microscopy. (Fig 8A and 8B, 0h) demonstrate that ~20% of cells in the pleomorphic bloodstream form culture induced for 24h to overexpress REG9.1 spontaneously expressed EP procyclin without exposure to the normal differentiation trigger, cis aconitate, whereas no cells with this phenotype were observed in uninduced cultures. When transferred to procyclic form culture media, the proportion of procyclic form cells progressively increased consistent with either further differentiation to procyclic forms with continued culture, or the viable outgrowth of the differentiated population (S4 Fig). The ability of REG9.1 to potentiate differentiation was not simply restricted to a founder population however; when bloodstream forms induced to overexpress *REG9*.*1* were exposed to the differentiation trigger cis aconitate, 60% of cells expressed EP procyclin within 4 hours, contrasting with less than 20% in uninduced cultures (p<0.05; Fig 8B). This demonstrated that they showed enhanced differentiation-competence. Thus, depletion of REG9.1 promotes the differentiation to a stumpy form transcriptome profile, whereas its overexpression potentiates differentiation to procyclic forms.

### *REG9*.*1* exhibits a differential cellular location in stumpy forms

**Fig 9:**
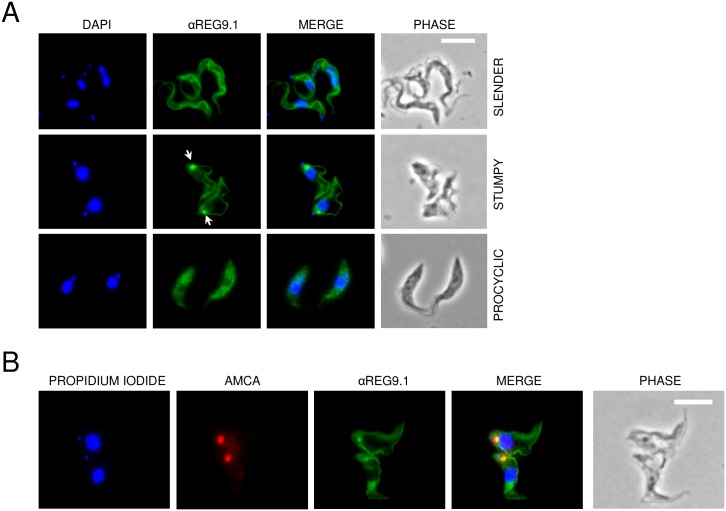
Differential cellular location of REG9.1 in the trypanosome life cycle. A. Cellular location of REG9.1 in slender, stumpy and procyclic forms. Kinetoplast and nuclear DNA was visualised with DAPI staining (pseudocoloured blue), whereas the cell outline was visualised via Phase contrast microscopy. The REG9.1 signal is cytoplasmic in slender and procylic forms but concentrated at the cell posterior in stumpy forms (arrowed). Bar = 10μm. B. Co staining of two stumpy cells with REG9.1 (Green), AMCA (Red, staining the flagellar pocket) and propidium iodide (pseudocoloured Blue, staining the nucleus and kinetoplast). The REG9.1 staining is close to but not coincident with the AMCA staining at the flagellar pocket.

Given the differential regulation by REG9.1 of stumpy-enriched transcripts, yet similar expression in slender and stumpy forms, we analysed the cellular distribution of REG9.1 in different developmental forms by immunofluorescence. Fig 9A demonstrates that in slender and procyclic forms REG9.1 was distributed throughout the cell body, consistent with a cytoplasmic location, although some flagellar staining was also observed in slender forms. In contrast, in all stumpy cells examined there was a concentration of signal close to the flagellar pocket of the cell, and also close to the cell flagellum (Fig 9A, arrowed). Co-staining of the cells with AMCA which stains the cell surface and flagellar pocket demonstrated that the REG9.1 signal was only partly overlapping with the flagellar pocket (Fig 9B) and ‘no first antibody’ controls confirmed the signal was not caused by secondary antibody recognition of host antibodies in the flagellar pocket. Since REG9.1 has been detected in procyclic form stress granules by proteomic analysis [34], we explored whether REG9.1 coalesced from its uniform cytoplasmic distribution in procyclic forms to discrete foci upon starvation or heat shock. Under starvation conditions (2h in PBS) REG9.1 co-localised partially with the stress granule marker Scd6 [35] (S5A Fig), but this was not evident with heat shock, where Scd6 signal was also not obvious. Interestingly, however, 20–25% of the heat shocked procyclic cells (and a smaller percentage of the starved cells) showed a concentration of REG9.1 close to the flagellar pocket, similar to that observed in stumpy forms (S5B Fig).

In summary, our genome wide analysis for regulators of stumpy-enriched gene expression has identified REG9.1 as a novel key regulator of development in *Trypanosoma brucei*. However, despite similar abundance in bloodstream form stages, REG9.1 shows differential location, being cytoplasmic in slender forms and concentrated at a location close to the flagellar pocket in stumpy forms. In procyclic forms, the cytoplasmic protein becomes concentrated in discrete foci upon stress.

### Discussion

We have exploited an unbiased genome-wide RNAi selective screen to identify regulators that repress the expression of transmission stage transcripts in proliferative bloodstream forms of the African trypanosome. This exploited our previous identification of the *ESAG9* family as a group of stumpy-enriched transcripts, whose protein products are potentially secreted during the differentiation from stumpy to tsetse midgut procyclic forms [18]. Several outputs from the selective screen were validated as gene regulators whose gene silencing by RNAi resulted in the derepression of the *NeoR* fused to the 3’UTR of one *ESAG9* gene copy. Of these, a predicted RNA-binding protein, *REG9*.*1*, regulated *NeoR* mRNA and protein levels as well as endogenous *ESAG9* transcript levels. Moreover, *REG9*.*1* depletion in pleomorphic trypanosomes resulted in premature development to the parasite’s transmission stage *in vivo*, independent of the parasites normal quorum-sensing response. With overexpression of *REG9*.*1*, differentiation of bloodstream form parasites to tsetse midgut procyclic forms was potentiated. Hence *REG9*.*1* influences the surface architecture of *T*. *brucei*, repressing transmission stage transcripts in slender forms and activating the expression of tsetse midgut form transcripts when expressed at the elevated level typical of procyclic forms. This implicates the molecule as an important regulator of the parasite’s development and cell surface protein expression, with differential effects dependent upon its expression level and life cycle stage.

*REG9*.*1* has been identified in previous global studies investigating the regulation of gene expression in trypanosomes. For example, the artificial tethering of a *REG9*.*1*-lambda-N peptide fusion to a reporter gene 3’UTR bearing a BoxB lambda-N recognition sequence resulted in reporter gene repression [27, 36] consistent with the repressive effect we observed on *ESAG9* transcripts. Intriguingly, however, we found that the overall abundance of *REG9*.*1* did not strongly differ between slender and stumpy forms, despite the differential regulation of transcripts such as *ESAG9* between these developmental forms. One explanation for this could be the evidence for post-translational modification of both the endogenous and ectopically expressed protein revealed by the migration of distinct isoforms when detected with *REG9*.*1* specific antibody. More obviously, however, the distinct cellular distribution of *REG9*.*1* in slender and stumpy forms could contribute, with the protein being concentrated at a posterior site in stumpy forms, but not slender or procyclic forms. Hence, sequestration of *REG9*.*1* in stumpy form cells might alleviate transmission stage mRNAs from repression, with *REG9*.*1* generating its effects through differential location rather than differential abundance in slender and stumpy forms. Interestingly, other identified regulatory components of stumpy formation (Tb927.11.6600, Tb927.11.2250) have been found coassociated in stress granules with REG9.1 [12, 34] and we observed a concentration of REG9.1 upon starvation and heat shock in procyclic forms. We suggest this differential location provides a mechanism for regulation via REG9.1 independent of the protein’s overall abundance, although the nature of the signal concentration close to the flagellar pocket awaits further cytological analysis. A posterior concentration of the XRNA stress granule marker near to the posterior location of procyclic form cells has been described previously [35] but we failed to detect signal when using a XRNA-YFP fusion cell line, precluding colocalisation analysis with REG9.1. Nonetheless, the cellular distribution reported for XRNA at the cell posterior [35] does not seem quite the same as the flagellar pocket proximal location we observed for REG9.1 in stumpy forms.

The sophistication of gene expression control in trypanosomes is highlighted by the complexity of regulatory signals and regulatory factors that act on distinct transcripts. For *ESAG9*, a previous mapping of signals in the 3’UTR that contribute to stumpy-enriched expression identified a short domain that contributed to expression in stumpy forms, but with more subtle effects in monomorphs [22]. We have confirmed regulatory control via this domain in our reporter experiments, since deletion of the element resulted in increased *NeoR* expression and reduced sensitivity to G418 (S2 Fig). However, *REG9*.*1* regulation of *NeoR* linked to the *ESAG9* 3’UTR was not lost upon deletion of this domain, demonstrating that regulation after REG9.1 depletion does not operate through this sequence region alone (S2 Fig). Similarly, a MEME search (http://meme-suite.org) of transcripts co-regulated with *ESAG9* mRNAs in our global transcriptome analysis identified short motifs enriched in the regulated transcripts distinct from the previously mapped regulatory element (AUGCGAAUAA and GAAAACAUGC/ GCGUACAUGA), but deletion of these motifs in the *ESAG9* 3’UTR did not consistently increase transcript expression when tested using a NeoR reporter system (S6 Fig). Hence, the 3’UTR sequences that determine the regulation of *ESAG9* when REG9.1 is depleted are expected to be multifactorial and dispersed. This is consistent with the analysis of 3’UTR regulatory signals across several studies where linear motifs have not been detected.

As well as *ESAG9*, the depletion of REG9.1 in bloodstream forms derepressed two previous uncharacterised protein families predicted to encode components of the trypanosome cell surface proteome, Family 5 and Family 7. Both of these families, like ESAG9, are *T*. *brucei* specific [21]. The coregulation of Family 5 and Family 7 members with ESAG9 suggests these molecules may have hitherto unexpected roles in the transmission biology of the parasite. Both have signal peptides for trafficking to the parasite surface and a transmembrane helix [21] and are of similar size (230–270 amino acids) but otherwise have no recognisable features that would allow functional assignment in the life cycle. They are also not detected in the proteomic analyses of stumpy forms and early differentiating cells [17] and therefore these proteins may not remain cell associated, similar to the extracellular release of ESAG9 members [18]. Intriguingly, the central coil-coil region of the regulated Family 7 members show some structural similarity (http://toolkit.tuebingen.mpg.de/hhpred) to the LRIM1 and APL1C regulators of the TEP1 complement-like protein in Anopheles mosquitos [37]. It will be interesting to explore whether these molecules have functional significance in transmission, for example by acting as countermeasures to arthropod immune factors when stumpy forms enter the tsetse fly.

The transmission biology of trypanosomes remains poorly understood. Nonetheless, the restriction of ESAG9, Family 5 and Family 7 proteins to *T*. *brucei* suggests a function related to the unique niche that the parasite invades upon entering the fly, or the specific biology of stumpy forms that are absent in *T*. *congolense* and *T*. *vivax*. Hence *REG9*.*1* may have specialised to influence the specific surface architecture of the *brucei* group of African trypanosomes, including *T*. *b*. *gambiense* and *T*. *b rhodiesiense* that cause sleeping sickness in humans, and is an example of a molecule that maintains the slender form whilst inhibiting progression to stumpy forms, a so-called ‘slender retainer’ [38]. In contrast, when overexpressed at levels normally seen in procyclic forms, REG9.1 drove the spontaneous differentiation of bloodstream forms to procyclic forms in a small proportion of cells, a phenotype opposing two identified negative regulators of differentiation, TbPTP1[39] and RDK1 [40]. Analysis of the REG9.1 overexpressing bloodstream forms demonstrated they also expressed procyclin on more cells in the population than uninduced cells when exposed to cis aconitate. This indicated that there was a higher proportion of differentiation-competent cells (i.e. intermediate or stumpy forms) after REG9.1 elevation, this being supported by the down regulation of the haptoglobin haemoglobin receptor mRNA, a hallmark of intermediate and stumpy forms [41]. PAD1, however, was not strongly elevated demonstrating that fully developed stumpy forms were not induced or did not persist prior to differentiation to procyclic forms. A dominant negative effect of REG9.1 overexpression can also not be excluded as a contributor to the phenotypes observed although we were careful to ensure expression levels were no higher than those seen normally in procyclic forms. Overall, our results highlight that the developmental capacity of trypanosomes can be governed by the expression level of individual regulators, with REG9.1 able to promote differentiation to procyclic forms from the bloodstream in the absence of external signals, whilst RBP6 [8] is able to promote differentiation between developmental forms in the tsetse fly.

To date there has been a significant focus on the role of VSG and procyclin as the major surface proteins of the bloodstream and procyclic forms, respectively. The co-regulation of ESAG9, Family 5 and Family 7 suggests there is significant further functional complexity to dissect with respect to the surface interactions of the parasite with its environment during transmission. The identification of REG9.1 as a global regulator of these surface protein families provides a tool to dissect the species-specific tropism of different African trypanosomes upon transmission and within their tsetse fly vector.

## Materials and methods

### Ethics statement

Animal experiments in this work were carried out in accordance with the local ethical approval requirements of the University of Edinburgh and the UK Home Office Animal (Scientific Procedures) Act (1986) under licence number 60/4373.

### Trypanosome culture, constructs and transfection

Monomorphic *T*. *brucei* bloodstream form 2T1:T7 [26] and 2T1 cells were used for the RNAi library screen [42] and for the individual validation of the screen hits, respectively. Pleomorphic *T*. *brucei* AnTat1.1 90:13 cells [43] were used for the phenotypic analysis of *REG9*.*1* in bloodstream forms. The 2T1:T7 and 90–13 cell lines express the T7 RNA polymerase and tetracycline repressor protein while the 2T1 line lacks the T7 RNA polymerase.

Slender forms were either grown *in vitro* or harvested from MF1 female mice at 3 days post infection. Stumpy forms were harvested from cyclophosphamide-treated MF1 female mice at 5 or 6 days post infection. All bloodstream form cell lines were grown in HMI-9 at 37°C in 5% CO_2_. Differentiation of bloodstream to procyclic forms was induced by addition of 6 mM cis-aconitate and a temperature change from 37°C to 27°C. For the phenotypic analysis of *REG9*.*1* in procyclic forms, AnTat1.1 90:13-*REG9*.*1* RNAi cell line was differentiated to procyclic forms and grown in SDM79 + 50mM N-Acetylglucosamine (GlcNAc) at 27°C in 5% CO_2_ [44]. Procyclic 29.13 cell line was used as a control, grown in the same conditions.

The *neo-ESAG9* 3’UTR reporter cell line and its variations were generated using the p5’neo5’ vector, that contains the *neo* gene flanked by the *aldolase* 5’ and 3’UTRs (derived from pbRn, [45], but with BLE resistance and the rRNA promoter removed). The different bloodstream form RNAi cell lines were generated using the stem loop vectors pRPa (for monomorphs) [46] and pALC14 (for pleomorphs)[47], linearized with AscI or NotI respectively, prior to transfection. RNAi target gene fragments were selected based on default settings of the RNAit software [48]. Overexpression constructs were generated using a modified pLEW100v5bld-BSD vector (containing the tagging TY/YFP/TY region derived from pDex577-Y). Monomorph and procyclic transfections were performed as described by [49]. Pleomorph transfections were done as described by [50]. Selection was applied by using the appropriate drugs: Geneticin (G418 1 μg/ml; for p5’neo5’), hygromycin (HYG 2.5 μg/ml; for pRPa^iSL^), puromycin (PURO 0.5 μg/ml; for pALC14), blasticidin (BSD 10 μg/ml; for pLEW100v5b1d-BSD) and phleomycin (BLE 1 μg/ml; for pDex577-Y).

### Genome wide selection

2T1:T7 cells were first transfected with pT7Pol.BSD (containing the T7 RNApol and replacing BLE resistance by BSD) to generate the parental cell line 2T1^T7^. 2T1^T7^ was then transfected with p’5neo5’-*ESAG9* 3’UTR (Long), where the *NEO* gene is flanked by 1057nt of the *ESAG9*-3’UTR, to generate 2T1^T7^/*E9*(Long). These cells were first selected with 1 μg/ml G418 and then sensitivity to higher doses of the drug was determined for each cell line. 2T1^T7^/*E9*(L) was subsequently transfected with pRPa^Sce^* and the obtained clones selected after 6 days and tested for PURO sensitivity (replaced by BLE after transfection). Parasites were finally induced with 1 μg/mL of tetracycline to induce the I-*Sce*I digestion and 3h later 40 μg of the RNAi library DNA were used to transfect 2x10^8^ cells. 6 h later transfected cells were selected with BLE. Two days post-transfection RNAi was induced with 1 μg/ml of tetracycline for 24h and subsequently divided in different flasks containing 35x10^6^ cells with different concentrations of G418 (10 and 25 μg/ml). A control was done with no tetracycline and no G418. After 10–15 days a selected population was obtained and its DNA extracted. Genomic DNA extraction, validation through independent RNAi constructs, Ion Torrent sequencing and bioinformatics analysis were done as described by [12].

### Antibodies and western blotting

Protein samples were boiled for 5 min in Laemmli loading buffer (except PAD1 samples, that remained unboiled), run in a 10 or 12% SDS/PAGE and transferred to a nitrocellulose membrane. After blocking with 5% milk in PBS-T (0.05% Tween in PBS) for at least 30 min at room temperature, membranes were incubated with antibodies overnight at 4°C under agitation in 5% milk in PBS-T. The primary antibodies were used at the following dilutions: αPAD1 (1:1000); αNeo (1:1000, Neomycin Phosphotransferase II, Merck-Millipore); αEF1α (1:7000, Elongation Factor 1-alpha, Merck-Millipore); α*REG9*.*1* (1:2500; polyclonal antibody generated by Eurogentec against the recombinant protein), αBB2 (1:5,[51]). After 3 washes in PBS-T, two different systems for antibody detection were used. Chemiluminescence required 1h incubation at room temperature with a horseradish peroxidase-conjugated secondary antibody in 5% milk (in PBS-T) that was either α-rabbit (1:5000) or α-mouse (1:5000). The membrane was then washed three times in PBS-T and revealed using Pierce ECL2 western blotting substrate (ThermoScientific). Alternatively, protein was visualised by incubating the membranes for 1h at room temperature with a secondary antibody conjugated to a fluorescent dye diluted 1:5000 in 50%Odyssey Blocking buffer/50% PBS-T and finally scanning the membrane with a LI-COR Odyssey imager system.

### Northern blotting and transcriptome analysis

For northern blotting, RNA preparation and analysis was carried out as described by [49]. For the transcriptome analysis, RNA was purified using the RNeasy purification kit (Qiagen) and sent to BGI Hong Kong. Quality of sequence data provided by BGI was assessed using FastQC (http://www.bioinformatics.babraham.ac.uk/projects/fastqc/). No additional processing of the primary data was required.

### Phenotypic analysis

For cell cycle analysis and flow cytometry, cell preparation and analysis was carried out as described by [12]. Growth curves were generated by analysing typically three independent flasks of the same cell line grown in parallel (biological replicates) for each clone. Starvation of procyclic cells was induced by washing the parasites twice in PBS and incubating them for 2h at 27°C in PBS. Heat shock was induced by incubating the parasites at 41°C for 1h. For REG9.1 localisation, the Sulfo-NHS-AMCA (0.75mM) probe for flagellar pocket staining was incubated with 4x10^6^ parasites for 10 min at 4°C and then washed twice with PBS. For immunofluorescence, two different approaches were used depending on the antibody used for the staining. For cell surface staining, cells were smeared on a glass slide, allowed to dry and fixed in cold MeOH for 20 min at -20°C and subsequently rehydrated in PBS. For internal staining and eYFP visualisation, cells were fixed at RT for 10 min with 4% paraformaldehyde and then permeabilized with 1% triton for 15 min. In both approaches cells were blocked with 2% BSA in PBS for 30 min at 37°C and incubated with either αEP1 1:250 and αVSG221 1:200 or αREG9.1 1:1000, all in 2% BSA in PBS for 45 min at 37°C. Then cells were washed twice in PBS and incubated in αmouse Alexa fluor 488 and αrabbit Alexa fluor 568 or 488, in 2% BSA in PBS for 45 min at 37°C. Subsequently, parasites were stained with either 10 μg/mL DAPI or Propidium Iodide in PBS and washed twice in PBS. Finally, slides were mounted with Mowiol containing 2.5% DABCO and analysed on a Zeiss Axio Imager Z2. QCapture was used for image capture.

### Accession numbers

The GEO accession number for the RNA Seq sequence data is GSE81765.

## Supporting information

S1 Table Ion torrent read density for genomic locations across the *T*. *brucei* genome after genome-wide RNAi selection using the long form *ESAG9* 3’UTR selection construct. *DRBD5*, *ZFP3* and *REG9*.*1* are highlighted.
(XLSX)

S2 Table RNA-seq analysis after REG9.1 depletion in pleomorphic cells. Sheet 1 shows the respective changes in transcript abundance with respect to uninduced cells; other sheets show the respective log FC change of different transcript families regulated by *REG9*.*1*. The regulation of the Family 2, 5 and 7 surface phylome families is shown on a histogram in chart “Fam charts”.
(XLSX)

S3 Table RNA-seq analysis after REG9.1 overexpression (through pLew ectopic expression) in pleomorphic cells. **Data availability**: Raw data for the data represented in S1, S2 and S3 Tables is available through the GEO database, with accession reference GSE81765.
(XLSX)

S1 Fig Validation of individual RNAi lines silencing genes selected in the RNAi library. A. Northern blots demonstrating the inducible gene silencing of *DRBD5* and *ZFP3*. Two independent RNAi clones were analysed for each (*DRBD5* clone 1 and 3; *ZFP3*, clone 1 and 2). B. G418 resistance (at 10μg/ml) of the respective RNAi clones for each target when RNAi were induced or not. In each case, RNAi results in decreased sensitivity to G418. Two different clones were used for each growth curve. For each clone, the curve was repeated at least twice (a representative experiment is shown), each time with three biological replicates. Error bars symbolize standard deviations of the biological triplicates. C. Northern blot of *NeoR* transcript levels when each RNAi line was induced or not; rRNA was used as a loading control. Clone 1 of the *DRBD5* RNAi lines exhibits far higher *NeoR* RNA than clone 3 for unknown reasons. D. Western blot of *NeoR* protein when each RNAi line was induced or not; EF1α was used as a loading control. E. Northern blot of *ESAG9* EQ transcript levels when each RNAi line was induced or not; rRNA was used as a loading control.
(TIF)

S2 Fig RNAi silencing of REG9.1 in the different reporter cell lines containing different length 3’UTRs of *ESAG9-EQ*. A. Reporter constructs with either a short form of the ESAG9 EQ 3’UTR (400nt) or with a previously characterised regulatory element deleted (Δe). B. a titration of G418 resistance for the NeoR gene flanked by the short form 3’UTR. Cells are less sensitive to G418 than when the NeoR is flanked by the long form 3’UTR. C., D. Northern blot of *NeoR* transcript levels when REG9.1 RNAi was induced or not in each reporter cell line; *NeoR* increases after REG9.1 depletion in both short form and mutant reporter cell lines. The increase in *NeoR* mRNA is more subtle than in the reporter cell line containing the 1057nt 3’UTR (Fig 2). rRNA was used as a loading control. Two independent RNAi clones were analysed for each reporter cell line: (2) and (4) for the short (400nt) 3’UTR and (1) and (2) for the short mutant (Δe) 3’UTR. For the reporter line with the short form of the ESAG9 3’UTR the endogenous levels of ESAG9EQ mRNA are also shown, confirming effective elevation of this transcript when REG9.1 is depleted.
(TIF)

S3 Fig **A**. DAPI and Phase contrast images of fields of bloodstream trypanosomes induced, or not, to silence *REG9*.*1* expression. Parasites are shown on day 3 and day 4 post-infection, with counterstaining with DAPI to reveal the kinetoplast and nucleus. The morphology of cells induced or not was equivalent despite the much lower parasitaemia of the induced cells. **B**. Cell cycle re-entry of parasites undergoing differentiation in response to cis aconitate. The profile of G1, and G2/M cells is shown as an average of both *REG9*.*1* RNAi R1 and R2 at 0h, 4h and 24h after exposure to cis aconitate. Control slender, stumpy and procyclic populations are also shown, with stumpy cells being arrested in G0/G1. An average of replicates R1 and R2 is shown, with errors bars symbolizing the standard deviations of both replicates. Analysis was carried by flow cytometry.
(TIF)

S4 Fig Ectopic overexpression of *REG9*.*1* potentiates differentiation to procyclic forms. Bloodstream form parasites are shown 24h after induction of overexpression, with parasites then being incubated in SDM79+GlcNAC, conditions that allow the continued survival and proliferation of differentiated procyclic forms. EP procyclin positive cells are detected in induced populations at low frequency (1–20%) 24 h after induction and accumulate with continued culture. Uninduced cells in the same conditions show many dead cells although differentiated cells also arise but at lower frequency and only after being in procyclic medium for 144 h.
(TIF)

S5 Fig **A**. REG9.1 partially colocalises with Scd6 into cytoplasmic foci after 2h starvation in procyclic forms. Procyclic forms were starved for 2h in PBS before fixing. Nuclear DNA was visualised with DAPI staining (in blue). The REG9.1 signal (in red) is distributed along the cytoplasm in untreated cells and concentrated in foci after starvation in PBS. Some of these foci co-localise (see arrows) with the stress marker Scd6 fused to YFP (in green). Bar = 10μm. **B**. REG9.1. location upon heatshock at 41°C in procyclic forms. The spot of REG9.1 staining close to the flagellar pocket is arrowed (a monochrome image is shown since this reveals the presence of the concentrated signal more clearly).
(TIF)

S6 Fig Effect of the deletion of identified motifs in the ESAG9 Long 3’UTR of the reporter construct. A. Schematic representation of the different deletions created in the ESAG9 Long 3’UTR. ΔE1 corresponds to the deletion of the Motif 1. ΔE1+E2 correspond to the deletion of both Motif 1 and Motif 2. B. Growth of the parasites containing the reporter gene flanked by the wild type, ΔE1 and ΔE1+E2 Long 3’UTRs of ESAG9 in the presence of different concentrations of G418; the deletion of the different motifs has no effect on the drug resistance.
(TIF)

S1 Supplementary data (REG9.1 RNAi—DOX): Video of the motility of procyclic forms not induced to knockdown expression of *REG9*.*1*. (MP4)

S2 Supplementary data (REG9.1 +DOX) Video of the motility of procyclic forms induced to knockdown expression of *REG9*.*1*. (MP4)
