# Supplementary figures and images for: Genome-wide RNAi selection identifies a regulator of transmission stage-enriched gene families and cell-type differentiation in Trypanosoma brucei
Source: PLoS Pathog. 2017 Mar 23;13(3):e1006279. doi: 10.1371/journal.ppat.1006279 (PMC5380359; doi:10.1371/journal.ppat.1006279)

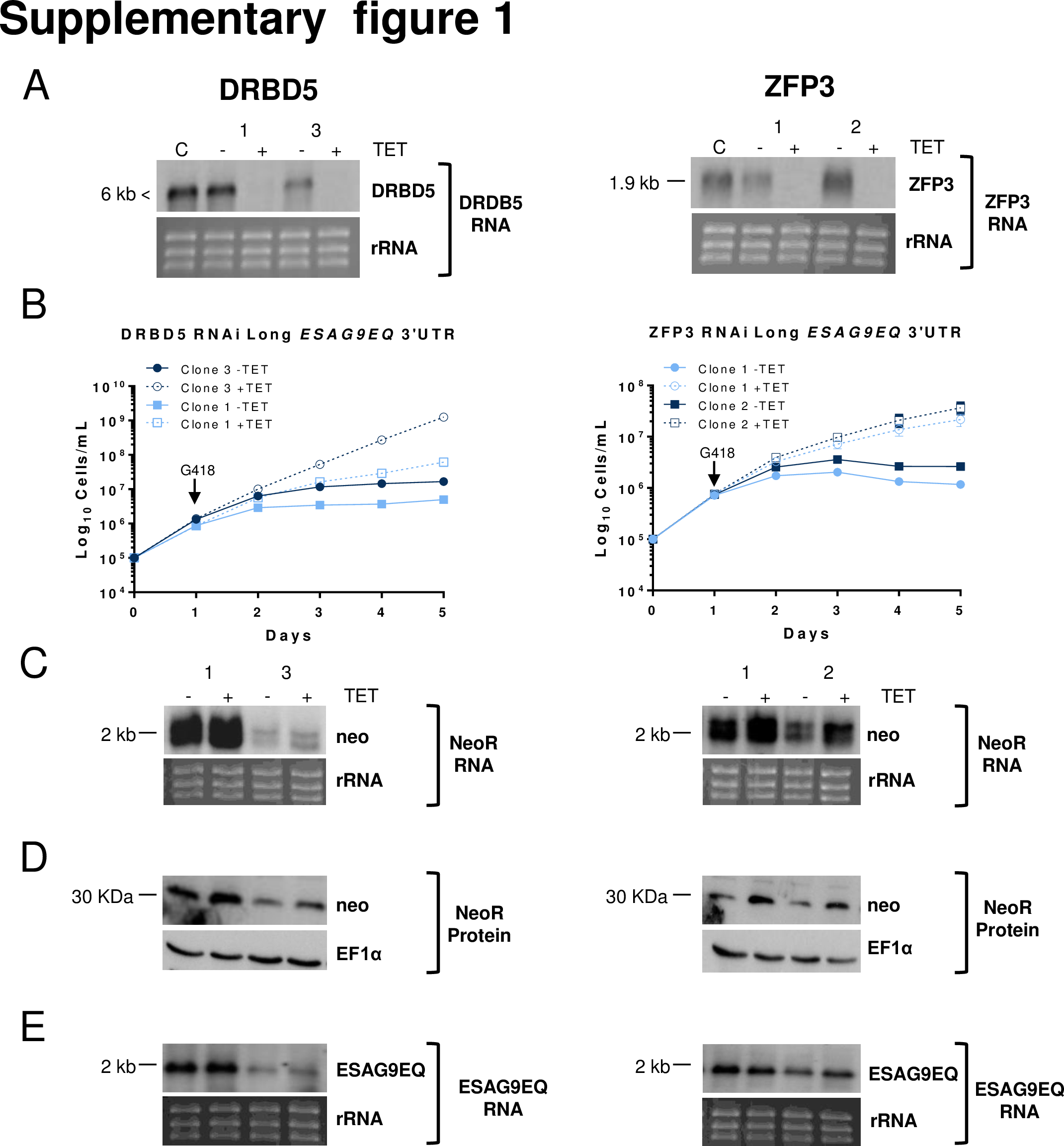

Supplement: S1 Fig — A. Northern blots demonstrating the inducible gene silencing of DRBD5 and ZFP3. Two independent RNAi clones were analysed for each (DRBD5 clone 1 and 3; ZFP3, clone 1 and 2). B. G418 resistance (at 10μg/ml) of the respective RNAi clones for each target when RNAi were induced or not. In each case, RNAi results in decreased sensitivity to G418. Two different clones were used for each growth curve. For each clone, the curve was repeated at least twice (a representative experiment is shown), each time with three biological replicates. Error bars symbolize standard deviations of the biological triplicates. C. Northern blot of NeoR transcript levels when each RNAi line was induced or not; rRNA was used as a loading control. Clone 1 of the DRBD5 RNAi lines exhibits far higher NeoR RNA than clone 3 for unknown reasons. D. Western blot of NeoR protein when each RNAi line was induced or not; EF1α was used as a loading control. E. Northern blot of ESAG9 EQ transcript levels when each RNAi line was induced or not; rRNA was used as a loading control. (TIF) [file ppat.1006279.s004.tif]

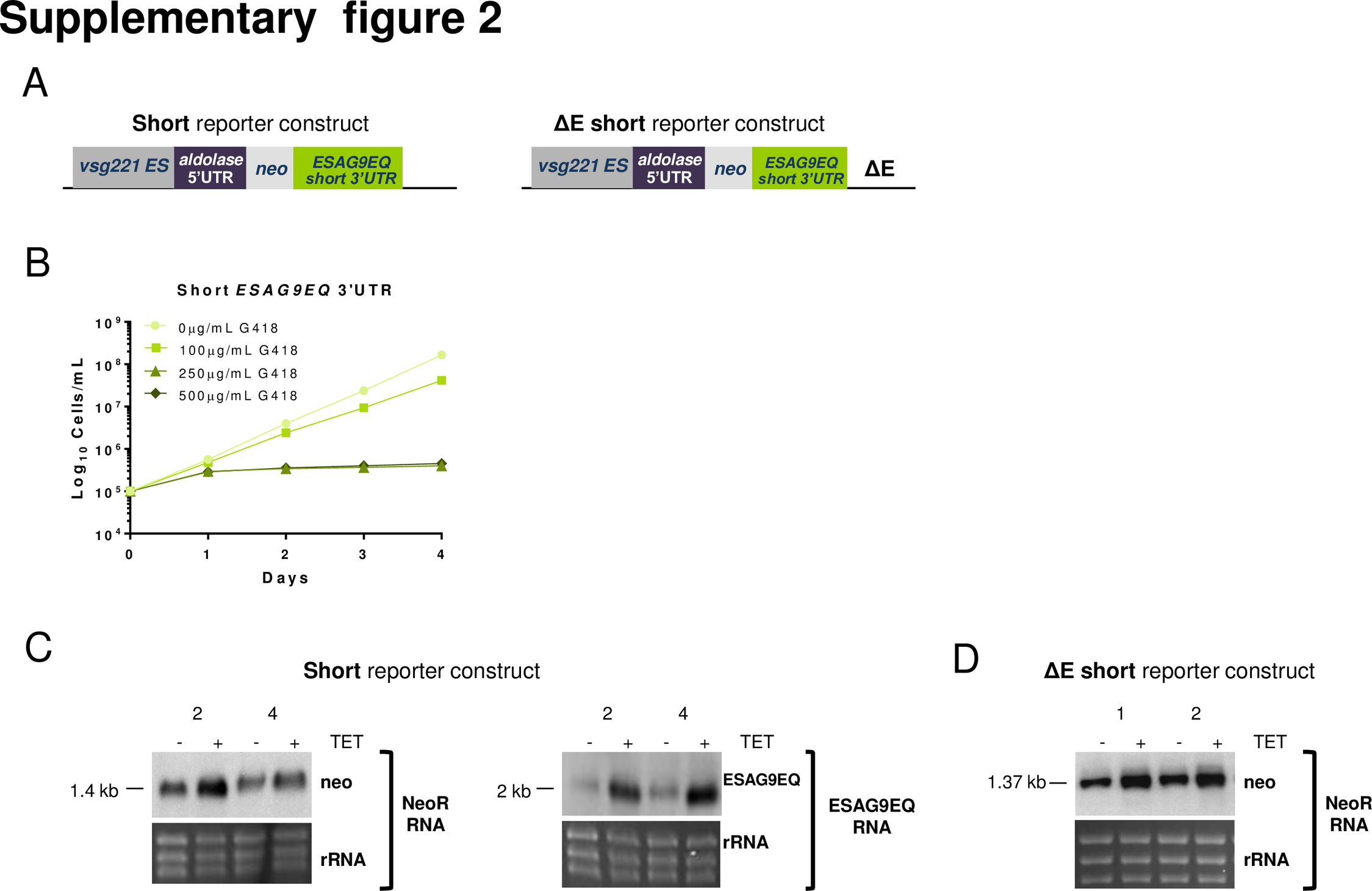

Supplement: S2 Fig — A. Reporter constructs with either a short form of the ESAG9 EQ 3’UTR (400nt) or with a previously characterised regulatory element deleted (Δe). B. a titration of G418 resistance for the NeoR gene flanked by the short form 3’UTR. Cells are less sensitive to G418 than when the NeoR is flanked by the long form 3’UTR. C., D. Northern blot of NeoR transcript levels when REG9.1 RNAi was induced or not in each reporter cell line; NeoR increases after REG9.1 depletion in both short form and mutant reporter cell lines. The increase in NeoR mRNA is more subtle than in the reporter cell line containing the 1057nt 3’UTR (Fig 2). rRNA was used as a loading control. Two independent RNAi clones were analysed for each reporter cell line: (2) and (4) for the short (400nt) 3’UTR and (1) and (2) for the short mutant (Δe) 3’UTR. For the reporter line with the short form of the ESAG9 3’UTR the endogenous levels of ESAG9EQ mRNA are also shown, confirming effective elevation of this transcript when REG9.1 is depleted. (TIF) [file ppat.1006279.s005.tif]

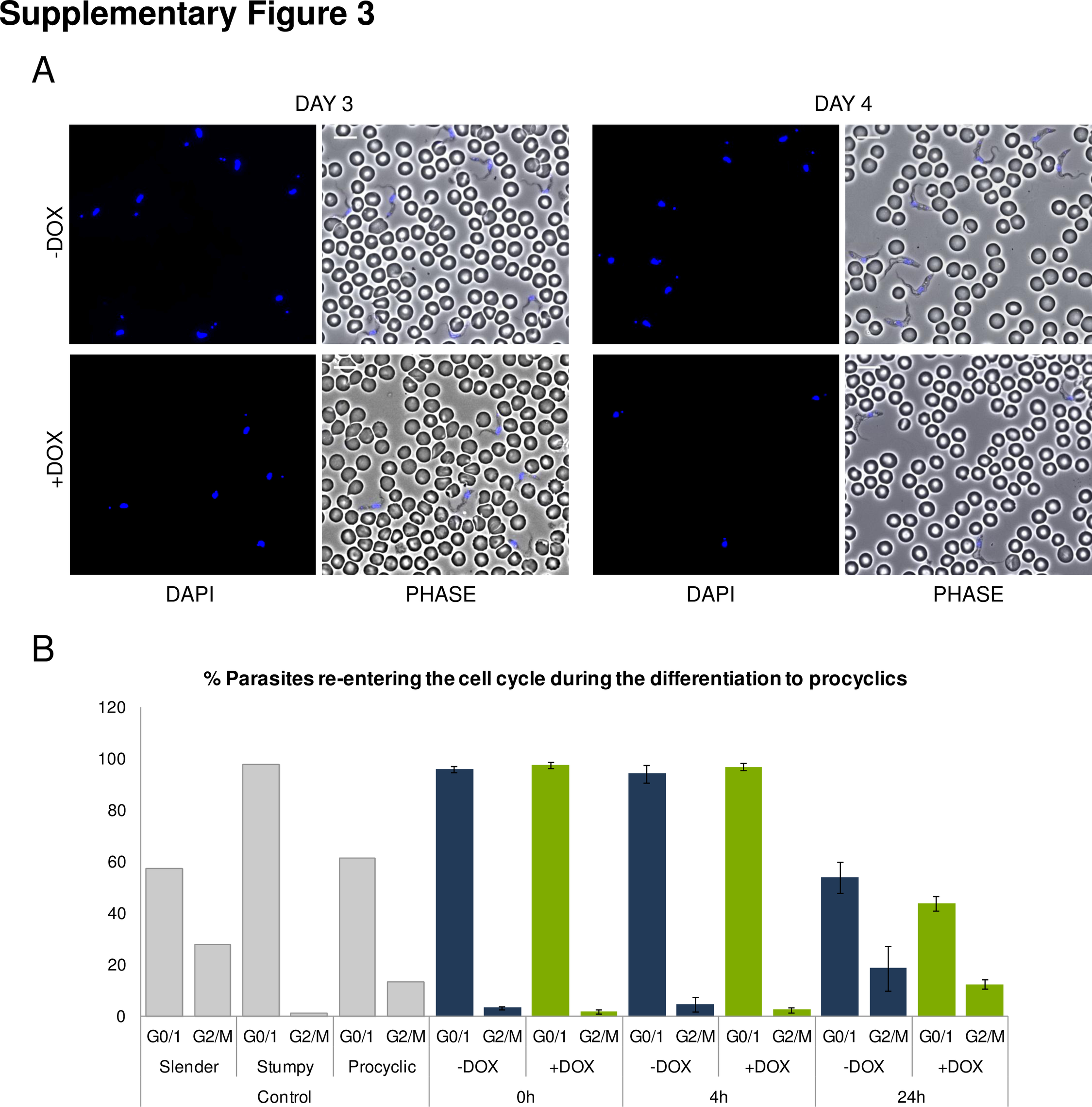

Supplement: S3 Fig — A. DAPI and Phase contrast images of fields of bloodstream trypanosomes induced, or not, to silence REG9.1 expression. Parasites are shown on day 3 and day 4 post-infection, with counterstaining with DAPI to reveal the kinetoplast and nucleus. The morphology of cells induced or not was equivalent despite the much lower parasitaemia of the induced cells. B. Cell cycle re-entry of parasites undergoing differentiation in response to cis aconitate. The profile of G1, and G2/M cells is shown as an average of both REG9.1 RNAi R1 and R2 at 0h, 4h and 24h after exposure to cis aconitate. Control slender, stumpy and procyclic populations are also shown, with stumpy cells being arrested in G0/G1. An average of replicates R1 and R2 is shown, with errors bars symbolizing the standard deviations of both replicates. Analysis was carried by flow cytometry. (TIF) [file ppat.1006279.s006.tif]

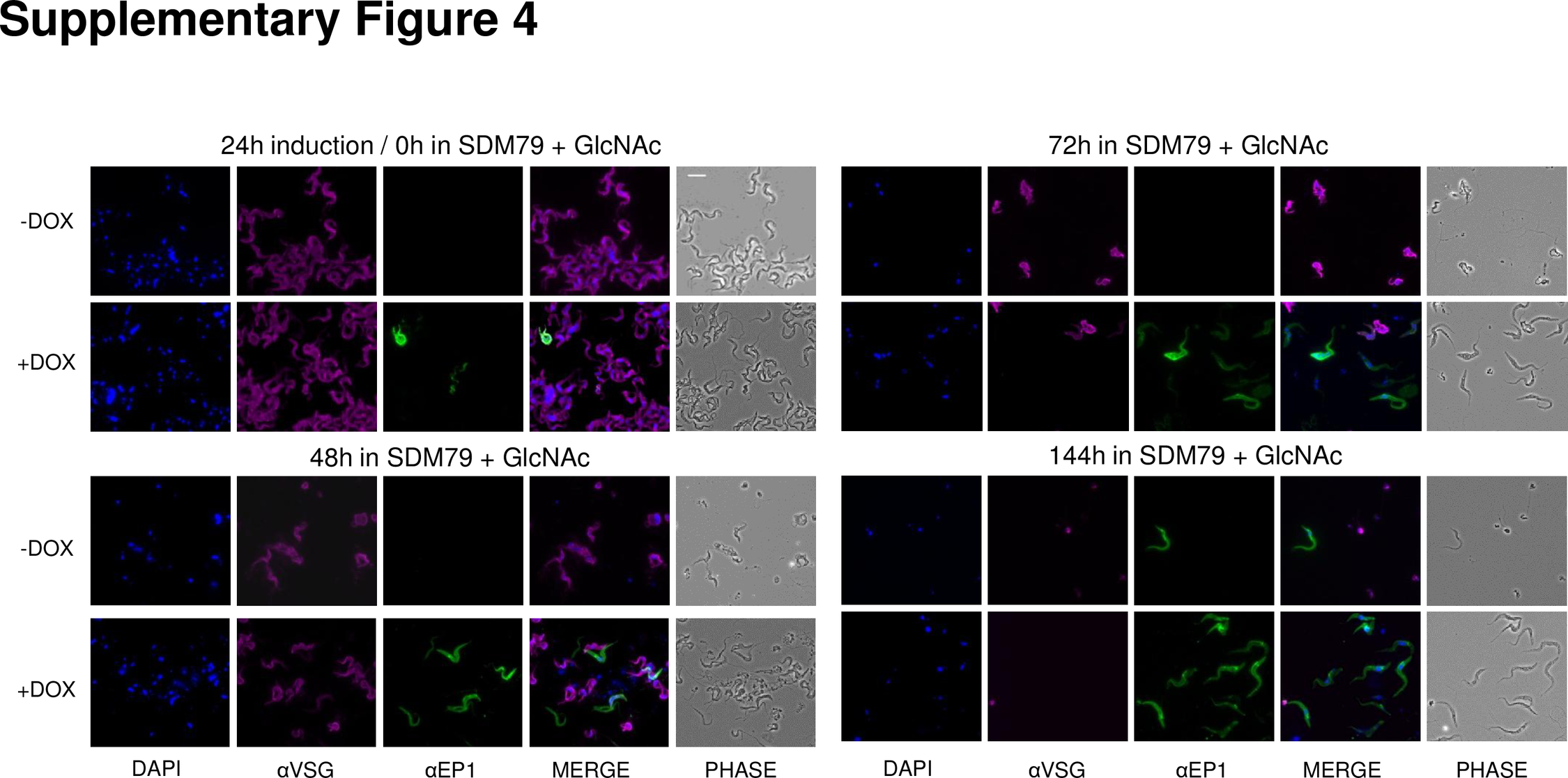

Supplement: S4 Fig — Bloodstream form parasites are shown 24h after induction of overexpression, with parasites then being incubated in SDM79+GlcNAC, conditions that allow the continued survival and proliferation of differentiated procyclic forms. EP procyclin positive cells are detected in induced populations at low frequency (1–20%) 24 h after induction and accumulate with continued culture. Uninduced cells in the same conditions show many dead cells although differentiated cells also arise but at lower frequency and only after being in procyclic medium for 144 h. (TIF) [file ppat.1006279.s007.tif]

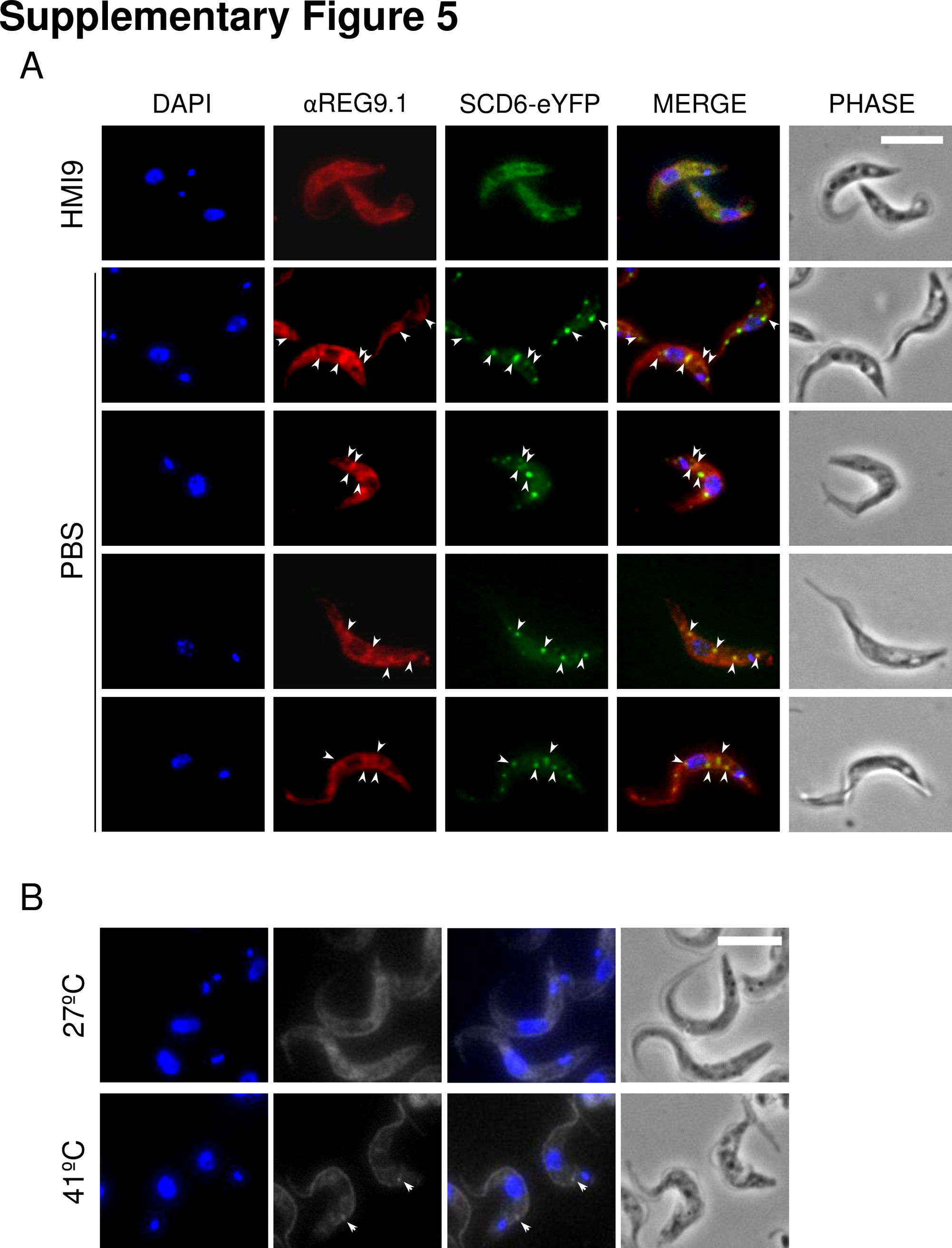

Supplement: S5 Fig — A. REG9.1 partially colocalises with Scd6 into cytoplasmic foci after 2h starvation in procyclic forms. Procyclic forms were starved for 2h in PBS before fixing. Nuclear DNA was visualised with DAPI staining (in blue). The REG9.1 signal (in red) is distributed along the cytoplasm in untreated cells and concentrated in foci after starvation in PBS. Some of these foci co-localise (see arrows) with the stress marker Scd6 fused to YFP (in green). Bar = 10μm. B. REG9.1. location upon heatshock at 41°C in procyclic forms. The spot of REG9.1 staining close to the flagellar pocket is arrowed (a monochrome image is shown since this reveals the presence of the concentrated signal more clearly). (TIF) [file ppat.1006279.s008.tif]

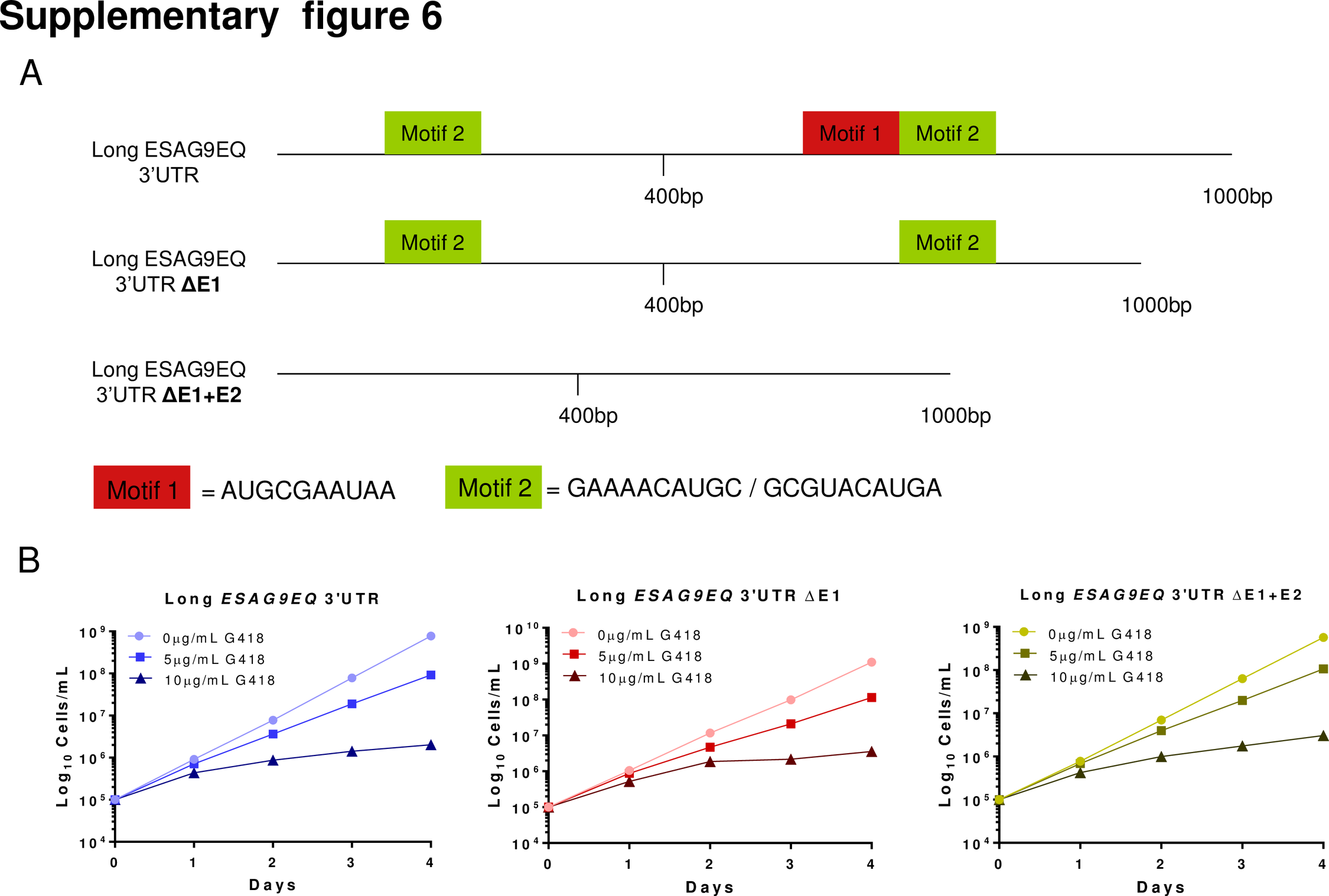

Supplement: S6 Fig — A. Schematic representation of the different deletions created in the ESAG9 Long 3’UTR. ΔE1 corresponds to the deletion of the Motif 1. ΔE1+E2 correspond to the deletion of both Motif 1 and Motif 2. B. Growth of the parasites containing the reporter gene flanked by the wild type, ΔE1 and ΔE1+E2 Long 3’UTRs of ESAG9 in the presence of different concentrations of G418; the deletion of the different motifs has no effect on the drug resistance. (TIF) [file ppat.1006279.s009.tif]
